# Sclerostoma Tetracanthus1Read at Chicago before the Illinois State Veterinary Medical Association.

**Published:** 1903-05

**Authors:** N. I. Stringer

**Affiliations:** Watseka, Ill.


### SCLEROSTOMA TETRACANTHUS.[1]

By Dr. N. I. Stringer,
WATSEKA, ILL.

In presenting this subject for your consideration I do not do so with the thought of adding anything new upon the life-history or anatomy of this parasite.

I appreciate listening to a scientific paper and its discussion; but I am not competent to write such a paper, so will endeavor to present something that I have seen in actual practice, which I hope will be of interest to my brother practitioners and possibly may assist them to correctly diagnose a peculiar case now and then that may in the past have been a puzzler.

It has been my fortune, or misfortune, to have seen a good many cases of death in the equine race caused by these parasites.

There does not seem to be much literature upon this subject. The ravages of this parasite have usually been attributed to the *Strongylus armatus.*

From what knowledge I have been able to gain regarding the two parasites macroscopically they look very much alike, but, as I understand, the armatus infect the bloodvessels, producing aneurisms, embolisms, and thrombosis, while the tetracanthus confine themselves wholly to the intestines.

Fröhner only makes a mere mention of them. Neuman does not give a very full description of them.

Dr. J. F. Winchester, in 1892, read a very extensive paper before the United States Veterinary Medical Association about the armed sclerotome (*Strongylus armatus*), but did not mention anything about the tetracanthus. This most instructive paper may be read by referring to page 288 of the *Proceedings of the United States Veterinary Medical Association*, 1892; also to page 359, *American Veterinary Review*, October, 1892.

[1] Read at Chicago before the Illinois State Veterinary Medical Association.

In the January, 1893, number of the *American Veterinary Review* is published a paper read before the Iowa State Veterinary Medical Association by Dr. G. L. Buffington upon the subject of this paper.

The description and round of life is given by Dr. Buffington as follows: "The *Sclerostoma tetracanthum* is one of the very small nematodes inhabiting the intestines of the horse. The body is slightly tapering anteriorly, of a reddish-brown color when preserved in alcohol, but while in the intestinal canal of the host the larger ones are of a bright red and the smaller ones a dirty white color. They are from one-fourth to three-fourths of an inch long, the females being a little larger than the males. The mouth is circular, with a salient rim that has a crown of triangular teeth, and outwardly six papillæ, two lateral, small, and on each side of them two others, conical and very prominent. The buccal capsule cylindrical. The caudal pouch of the male is simply excised on the ventral surface. The posterior lines are trifurcated, the middle doubled, and the anterior cleft. In the female the tail terminates in a point, and the vulva is very near the anus. The digestive canal is complete. The ova are segmented in the uterus. They are laid in the intestine of the host, passed out with the feces, and if the proper conditions, warmth and moisture, are met with will hatch out in a few days. The external phases in their development are, according to Bailliet, analogous to those of the *Strongylus armatus*, and are about as follows: If after the ova are hatched out they gradually grow, their integument becomes folded and forms a kind of sheath in which the worm moults. It is at this period they enter the body of the host in the water the animal drinks, or, perhaps, on the green forage when on low, damp ground, pass into the intestines, and it is probable that they encyst themselves directly into the mucous membrane of the colon and cæcum without penetrating the circulatory apparatus. At least, no wandering parasites of this kind have ever been observed. They remain embedded beneath the mucous membrane until they attain sexual maturity, when they again enter the intestinal canal to pass the remainder of their lives."

I think the above quotation from Dr. Buffington gives about all that is known of the life-history of these parasites.

About 1891 or 1892 I had my first experience with the *Sclerostoma tetracanthum*. A Mr. M., living near Fairbury, Illinois, where I was practising at that time, called me to see two yearling colts that were acting very strangely, and, as he thought, were starving, as he had just brought them home from a hired pasture. I think it was some time in June or July. They seemed to be very weak and could hardly walk, and could only arise with much difficulty when down. They were somewhat emaciated. One died in a few hours after I saw them, and the other died a day or two after the first one. I was somewhat puzzled, but I always take the opportunity to hold a post-mortem whenever a case terminates "fatally," especially those of a peculiar nature. The large colon and cæcum contained uncountable numbers of the parasites, they were found adhering loosely to the mucous membrane. That same season I saw in that vicinity six or eight cases ranging from yearlings to five-year-olds. The animal usually dies in from a few hours to three or four days after they show signs of the trouble.

The usual symptoms first noticed are a weak, staggering gait and haggard expression. When forced to walk it seems to completely exhaust them. They usually quit eating; in some cases that I have seen they would attempt to eat and drink, but could not swallow either food or water, there seeming to be a paralysis of the entire alimentary tract. Occasionally one will go down shortly after showing symptoms, and become delirious; struggling and frothing at the mouth, I think, is due to the inability to swallow the saliva. Those of the weak, trembling, staggering gait will show pallor or blueness of the visible membranes. Those that are struggling and delirious may show a livid color of the membranes. Feces in most cases scant, urine of a very high color, usually of a very dark red, and in some cases almost black.

*Post-mortem.* Tissues almost bloodless; when large blood-vessels are severed blood flows like water, is very dark, almost black in color; it seems to be entirely defibrinated, no coagulum exists anywhere. Bowels very pale in color, in some cases small hemorrhagic spots may be seen dotted over the peritoneal surface of the large colon and cæcum; apex of cæcum may be considerably congested and gangrenous. In two or three cases I have seen perforations of the bowel, allowing the fluids to escape into the peritoneal cavity, causing peritonitis, the animals showing symptoms of a severe colic before death.

I will cite a few cases that I have had to deal with:

August 23, 1899, about sundown, I was called to Mr. B.'s, near Eureka, Illinois. On my arrival I found a mare lying on the ground on her side, spasmodic paroxysms of struggling, and pawing with forefeet, hind-limbs motionless, these paroxysms taking place every few minutes. She would pay no attention to the stroke of the whip or being spoken to. Temperature, 101° F.; respiration, 30; pulse, 60. She died next day, 24th, about noon. On the same evening when called, 23d, a horse, four years old, was lying down in the barn. I gave him a stimulant; he got up in about an hour. We then got him out of the barn; before morning he also went down, and could not get up; soon became delirious, and grew worse very fast. We killed him the next afternoon.

On the same evening (23d) found two mares, staggering gait, walked with much difficulty, unable to swallow anything. I gave each a capsule of aloin, but do not think it reached the stomachs. One of the mares died on the night of the 25th, and the other about five days later; her exit was hastened by the administration of a lot of concoctions during my absence by the owner and an old moss-backed empiric. On account of the animal not being able to swallow, the dope naturally found its way into the lungs, producing inhalation pneumonia.

The empiric's diagnosis of the trouble was "pizen" (poison); the owner contended that it was witches that was doing the mischief. You may imagine how I enjoyed the situation; the owner would not believe my diagnosis, neither would he discharge me, but insisted upon my staying on the field. In the first three cases upon post-mortem the mucous membranes of the large colon and cæcum were covered with the *Sclerostoma tetracanthum*. In the last case, owing to the decomposed condition of the animal, they seemed to have been destroyed. The lungs were gangrenous.

There were six horses in the barn, and they seemed to be feeling well except one, that was showing slight symptoms of trouble. I gave all six ol. lini. and spirits terebinth., followed in thirty-six hours with aloin; also ferri sulphide and nux, twice a day.

On the morning of September 1 the horse that was not feeling well went down and could not get up. He was a driving horse, five years old. I raised him with a sling, by the aid of which he was able to stand, drank water, and ate some food, being able to swallow, which none of the other affected ones could do. Before slinging him up he was pawing and showing similar symptoms to the others, but not so severe. They let him out of the sling in three or four days, when he went down, and the same symptoms returned. They again raised him, when he seemed to be all right. He got out of the sling twice after that during the next two weeks, when the same symptoms would return. In six weeks he was turned to pasture and showed no more signs of the trouble. The funny part of the programme was that just before the horse was turned to pasture the owner consulted two lady clairvoyants in Peoria, who agreed with him that all his trouble was caused by a certain man, who was his enemy, working under the power of the witches, and for a certain consideration they could break his charm. The owner accepted their proposition, went home, and took the horse out of the sling and let him go. But I cannot help but give the credit to the sling and the nux and iron.

One very peculiar thing about the effects of these parasites upon some animals is, where the symptoms develop slowly and the animal will partake of food and water, if they are kept on their feet by the aid of a sling and treated with vermifuges and tonics a good many of them will recover.

Six years ago I saw some cases near Fairbury, with Dr. Presler, for Mr. Pense, seven dying out of the twenty head of young horses in that pasture. Dr. Presler reports that last summer a year ago in that same neighborhood one man lost thirteen head of young horses. A year ago last September, in Dr. Presler's absence, I was called to the same locality to see some work horses that were affected with the tetracanthus. The man lost three out of the four that he owned.

In September, 1900, a Mr. G., near Milford, Illinois, lost five out of six head of horses, ranging from five to fifteen years old. Two of these were mares with colts by their sides. One mare lived about eight days after she began to show symptoms of the trouble. Part of the pasture that these were in was a woods pasture that had recently been used for hogs, and there were a good many hog wallows filled with water.

This year I have seen four cases. One yearling, near Woodland; post-mortem showed cæcum and large colon filled with feces, mucous membrane and feces covered with tetracanthus, apex of cæcum much congested and about a pint of the long white worm (*lumbricoides*) packed in it.

I was called to Cissna Park to see one case, a very large suckling colt, five months old; it died a half-hour before my arrival; held post-mortem; conditions same as above cases, except there was no congestion of apex of cæcum or lumbricoides present at that point.

On the fourth of last month (November) I was called six miles east of Watseka to see a yearling colt that was staggering about, showing the same symptoms as its mate, so the owner told me, that died about three days before. We found this one lying down when I got there; we assisted it to arise, but it walked about with much difficulty; it voided urine soon after it got up; urine a very dark red. It soon laid down, and did not get up again. It died next day about nine o'clock. I held autopsy, and I have never seen as many tetracanthus in any one case as I saw in this one. These two colts were alone in a pasture that was partly woods and some of it low along a creek.

The horses that died at Eureka were watered from a shallow well and dipped nearly dry every day.

All other cases that I have spoken of were in pastures that were low or where water stood in puddles; some were partly woods.

